# Supplementary material for: Serpentoviruses Exhibit Diverse Organization and ORF Composition with Evidence of Recombination
Source: Viruses. 2024 Feb 18;16(2):310. doi: 10.3390/v16020310 (PMC10892116; doi:10.3390/v16020310)
Supplement: Supplementary file 1 [file viruses-16-00310-s001.zip › SupplementalTables.pdf]

Supplemental Table 1. Primers and conditions used for rtPCR to bridge gaps between Illumina MiSeq next-generation sequencing fragments of novel serpentoviruses.

Virus	Sense Primer	Antisense Primer	Amplicon Size	Annealing Temp (◦C)
[OR131601-602] Green Tree Python Isolate	GT1132F (5′- TCA CAA GAG TCG TCA AGG GC-3′)	GT1911R (5′- CCG ACT CGG TGG CTA AAA CT-3′)	600	56
	GT3104F (5′- TGC CTG TAA AGA CAA CGG GAA-3′)	GTDegRev1 (5′- CAN NAA UGU UGG CCU CUC CA-3′)	400	59
[OR131606-607] Papuan Python	Pap16108F (5′-GGC CAC ACT ACA ACC GTG AT-3′)	Pap16656R (5′- GGG AGT TGT TGA TGC GTT GG-3′)	560	56
	Pap18966F (5′-TGG CTC GTG CTT GTA GGA AG-3′)	Pap19417R (5′- TGC GAC ATT CGT GAG GTC TT-3′)	330	55
	Pap24006F (5′-TTG AGT TGT CCT GAA GTG ACA-3′)	Pap24231R (5′- AGC TAA GTC AGG TGA TGG TGT-3′)	200	52
[OR131601-602] Bredl's Python	BR5986F (5′-TCA GAA GAC CAG GAC CAT GA-3′)	BR6212R (5′-TGG TGT TGC AGT TGC TGT TG-3′)	113	54
	BR25512F (5′-AGT AAG CAG GAA GAG CTC TGT-3′)	BR25842R (5′-TCG GCA TCC ACT TGA CTA AGA-3′)	300	54

Supplemental Table 2. Overview of nidovirus genomes used in serpentovirus genome characterization. Genbank accession numbers are organized by viral genus or subgenus as necessary. Associated hosts and genome overview are given for each viral group. The length of each genome section in kilobases is shown on the top line for each genome section. The second line for Open Reading Frames (ORFs) sections show the corresponding coded protein, labeled with ORF number as a subscript. Proteins common between all viruses are italicized. Untranslated Regions (UTRs) are labeled with the coronavirus genera (α, β, γ or δ) that modeled an appropriate UTR region structure using serpentovirus UTR sequence.

**Genus (Subgenus)**	**Accession #**	**Hosts**	**5′ UTR**	**ORF 1a**	**ORF 1b**	**ORF 2**	**ORF 3**	**ORF 4**	**ORF 5**	**ORF 6**	**ORF 7**	**ORF 8**	**3′ UTR**
Pregotovirus	OR131642-48,<br>MN161560,<br>OR131621-22,<br>MN161563,<br>MN161558,<br>MF351889,<br>MK722372,<br>MN161571,<br>MG752895,<br>KJ541759,<br>KJ935003,<br>OR131594-600,<br>OR131641,<br>MN161567,<br>MN161559,<br>OR131631-40,<br>MN161564,<br>MK182566,<br>OR131623-30,<br>MK722380,<br>MK722365,<br>MN161560,<br>MN161569,<br>OR131601-2,<br>MK722363,<br>MN161568,<br>MK722374,<br>MK722378,<br>MK722371,<br>MK722369,<br>MK722368,<br>MK722370,<br>MK722367,<br>MK722366,<br>MK722364,<br>MK722375,<br>MK722373,<br>MN161565,<br>OR131618-20,<br>OR131606-7,<br>MF685025,<br>KX184715	Pythons,<br>Skink, Turtle	1<br>β	15-18<br>*PP1A*	7<br>*PP1B*	2.8<br>$S_{(2)}$	0.8<br>$\mathrm{TM1}_{(3)}$	0.7<br>$M_{(4)}$	0.5<br>$N_{(5)}$	0.9-1.2<br>$\mathrm{TM2}_{(6)}$	1.5<br>$\mathrm{GP1}_{(7)}$	-	0.9<br>α & γ

Lyctovirus<br>(N/A)	MZ971349,<br>MZ971342,<br>MZ971345	N.Am.<br>Colubrids	1<br>β	14.5<br>*PP1A*	7<br>*PP1B*	2.9<br>$S_{(2)}$	0.7<br>TM1$_{(3)}$	0.7<br>$M_{(4)}$	0.5<br>$N_{(5)}$	2<br>GP2$_{(6)}$	-	-	0.8<br>γ
Lyctovirus<br>(Rebatovirus)	MG600030,<br>MG600029	Chinese<br>Colubrids,<br>Nematode	0.6<br>β	15.5<br>*PP1A*	6.9<br>*PP1B*	2.9<br>$S_{(2)}$	0.6<br>TM1$_{(3)}$	0.2-0.4<br>VP7$_{4}$/<br>VP13$_{4}$	0.7<br>$M_{(5)}$	05<br>$N_{(6)}$	2<br>GP2$_{(7)}$	-	0.6<br>γ
Lyctovirus<br>(Chalatovirus)	MT997160	Chameleon	0.3<br>β	17.2<br>*PP1A*	7<br>*PP1B*	2.9<br>$S_{(2)}$	1.4<br>VP54$_{3}$	0.8<br>$M_{(4)}$	0.5<br>$N_{(5)}$	0.9<br>VP33$_{6}$	0.3<br>VP12$_{7}$	0.2<br>VP7$_{8}$	0.6<br>-
Vebetovirus	MT997159	Chameleon	0.4<br>β	19.7<br>*PP1A*	7<br>*PP1B*	3.4<br>$S_{(2)}$	0.3<br>VP12$_{3}$	0.9<br>VP34$_{4}$	1.4<br>$M_{(5)}$	0.7<br>$N_{(6)}$	-	-	1.4<br>γ
Sectovirus	NC043490,<br>MG600031	Chinese<br>Colubrids	-	>9.7?<br>*PP1A*	6.9<br>*PP1B*	3.1<br>$S_{(2)}$	0.8-0.9<br>TM1$_{(3)}$	1-1.1<br>VP38$_{4}$/<br>VP43$_{4}$	0.6<br>$M_{(5)}$	0.5<br>$N_{(6)}$	1.5<br>GP1$_{(7)}$	1<br>VP37$_{8}$	0.9<br>γ
Septovirus	OR131608-17,<br>MN161566,<br>MZ971330-40,<br>MZ971304-5,<br>MZ971310-11,<br>MZ971279,<br>MZ971293,<br>MZ971285,<br>MZ971299-300,<br>MZ971286<br>OR131604-5	Pythons	1.2<br>β	~13.6<br>*PP1A*	6.5<br>*PP1B*	2.4<br>$S_{(2)}$	0.8<br>TM1$_{(3)}$	0.6<br>$M_{(4)}$	0.4<br>$N_{(5)}$	0.2-0.3<br>VP8$_{6}$/<br>VP13$_{6}$	-	-	1.1<br>α & γ
Infratovirus	MZ971343,<br>MN161572,<br>KC883638,<br>KX883638,<br>MG600028	Colubrids,<br>Nematode	1-2.3<br>β	~15<br>*PP1A*	6.8<br>*PP1B*	2.8<br>$S_{(2)}$	1.4<br>TM1$_{(3)}$	0.2-0.4<br>VP7b$_{4}$/<br>VP10$_{4}$/<br>VP13$_{4}$	0.6-0.7<br>$M_{(5)}$	0.5<br>$N_{(6)}$	-	-	0.9<br>α & γ
Sertovirus	MN161561,<br>MN161562	Tree Boa	0.4<br>β	13.5<br>*PP1A*	6.8<br>*PP1B*	3.2<br>$S_{(2)}$	0.6<br>TM1$_{(3)}$	0.6<br>$M_{(4)}$	0.4<br>$N_{(5)}$	-	-	-	1.6<br>-
Bostovirus*	NC027199	Bovine	0.5<br>β	9.3<br>*PP1A*	6<br>*PP1B*	1.7<br>$S_{(2)}$	0.6<br>$M_{(3)}$	0.5<br>$N_{(4)}$	1.3<br>GP2$_{(5)}$	0.2<br>VP9$_{6}$	-	-	0.3<br>γ
Bafinivirus*	NC038295	Minnow	0.8<br>β	~14.5<br>*PP1A*	7<br>*PP1B*	3.6<br>$S_{(2)}$	0.7<br>$M_{(3)}$	0.5<br>$N_{(4)}$	-	-	-	-	0.2<br>γ
Oncotcha-<br>virus*	NC026812	Salmon	0.8<br>β	~14.5<br>*PP1A*	7<br>*PP1B*	3.6<br>$S_{(2)}$	0.7<br>$M_{(3)}$	0.5<br>$N_{(4)}$	-	-	-	-	0.2<br>γ
Torovirus*	LC088094	Bovine	0.7<br>β	13.2<br>*PP1A*	6.8<br>*PP1B*	4.7<br>$S_{(2)}$	0.7<br>$M_{(3)}$	1.2<br>HE$_{(5)}$	0.3<br>$N_{(5)}$	-	-	-	0.2<br>α

*Non-Serpentovirus Outgroup

Common Proteins:
PP1A = Polyprotein 1A
PP1B =Polyprotein 1B
S = Spike
M =Matrix
N =Nucleoprotein
